# ALDOC modulates astrocytic glycolysis and AMPK/mTOR/HIF-1α signaling in Alzheimer’s disease

**DOI:** 10.3389/fnins.2026.1847340

**Published:** 2026-06-04

**Authors:** Zhenyuan Zhang, Yanmei Bai, Xiangjian Zhang, Jian Zhang, Guofeng Yang

**Affiliations:** 1 Department of Neurology, The Second Hospital of Hebei Medical University, Shijiazhuang, Hebei, China; 2 Hebei Key Laboratory of Vascular Homeostasis and Hebei Collaborative Innovation Center for Cardio-Cerebrovascular Disease, Shijiazhuang, Hebei, China; 3 Department of Neurology, The First Central Hospital of Baoding, Baoding, Hebei, China

**Keywords:** aldolase C (ALDOC), Alzheimer’s disease (AD), astrocytes, glycolysis, hypoxia-inducible factor-1α (HIF-1α), metabolic regulation

## Abstract

**Aims:**

Astrocytes provide crucial metabolic support for neurons and undergo significant metabolic changes in Alzheimer’s disease (AD). Aldolase C (ALDOC), an astrocyte-enriched glycolytic enzyme, may play a role in this process. This study aimed to investigate whether ALDOC modulates astrocytic metabolism to support neuronal energy supply in patients with AD and to assess its therapeutic potential.

**Methods:**

Hippocampal and cortical tissues from 6-month-old APP/PS1 and wild-type mice were subjected to western blotting, qPCR, and immunofluorescence staining for ALDOC and glycolytic proteins. An *in vitro* AD model was created using oligomeric *β*-amyloid (oAβ)-treated SVGp12 astrocytes. ALDOC was overexpressed or knocked down via plasmid or siRNA. Downstream effects on AMPK/mTOR/HIF-1α signaling and the expression of glycolytic markers (LDHA and PKM2) were evaluated by western blot and qPCR, as well as by lactate/ATP assays and extracellular acidification rate (ECAR) measurements. Neuron–astrocyte interactions were assessed in an SVGp12/SH-SY5Y coculture. Furthermore, the ability of magnesium ions to restore ALDOC expression was tested.

**Results:**

ALDOC was specifically expressed in astrocytes but was downregulated in APP/PS1 mice, accompanied by reduced HIF-1α and LDHA levels, suggesting glycolytic impairment. Similar downregulation occurred in oAβ-treated SVGp12 cells. ALDOC overexpression was associated with altered AMPK/mTOR/HIF-1α signaling, enhanced glycolysis, and increased lactate and ATP production, whereas its knockdown had the opposite effects. These outcomes appeared to depend on HIF-1α, as suggested by the rescue experiments. In coculture, ALDOC overexpression in astrocytes supported neuronal metabolic function. Moreover, magnesium ions restored ALDOC activity and glycolysis in oAβ-treated astrocytes.

**Conclusion:**

These results suggest that ALDOC is downregulated in APP/PS1 mice and is associated with glycolytic impairment. In oAβ-treated astrocytes, ALDOC appears to regulate glycolysis through the AMPK/mTOR/HIF-1α axis and may support neuronal energy via the lactate shuttle. Magnesium ions appear to offer a potential strategy for addressing the metabolic deficits in AD.

## Graphical abstract

**Figure:**
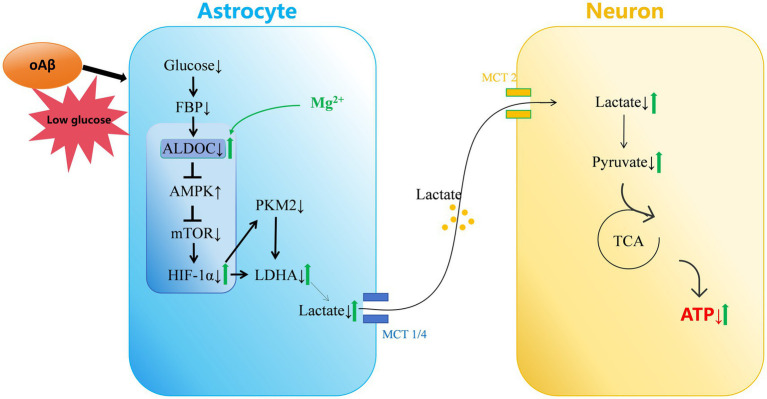
Proposed mechanistic diagram depicting how ALDOC modulates astrocytic glycolysis in response to oAβ, with a potential role for the AMPK/mTOR/HIF-1α pathway. The diagram also illustrates the protective role of Mg^2+^, which is shown to upregulate ALDOC, thereby counteracting the oAβ-induced downregulation of ALDOC and restoring astrocytic glycolytic flux and neuronal ATP generation.

## Introduction

Alzheimer’s disease (AD) is a progressive neurodegenerative disorder characterized by cognitive decline and behavioral dysfunction (Rostagno, 2022; Scheltens et al., 2021). Neuropathologically, AD features extracellular *β*-amyloid (Aβ) plaques and intraneuronal neurofibrillary tangles (Mucke, 2009). Neuronal loss and synaptic dysfunction directly underlie the clinical symptoms of AD. In parallel, neuronal function is intimately regulated by glial cells, particularly astrocytes, which support neuronal metabolism, synaptic plasticity, and immune surveillance (Mucke, 2009; De Strooper and Karran, 2016).

Emerging evidence suggests that AD involves chronic metabolic dysregulation (Preeti et al., 2022; Batra et al., 2023; Amin et al., 2025; Varma et al., 2018). The brain is highly energy dependent and relies primarily on glucose. Unlike adult neurons, which predominantly use oxidative phosphorylation (OXPHOS) for ATP production, astrocytes favor the generation of lactate by glycolysis, which serves as an energy substrate for neurons (Shichkova et al., 2024; Bonvento and Bolaños, 2021; Tang, 2020). Under pathological conditions such as AD, astrocytes undergo metabolic changes, but chronic stimulation leads to glycolysis deficits, impaired lactate production, and ultimately neuronal energy failure (Qian et al., 2023; de Sánchez Muniain et al., 2025).

The aldolase family (ALDOA, ALDOB, ALDOC) catalyzes the reversible cleavage of fructose-1,6-bisphosphate (FBP) into glyceraldehyde-3-phosphate and dihydroxyacetone phosphate, a critical branching point in glycolysis (Xiong et al., 2022; Zhang et al., 2014). ALDOC, a brain-specific isoform highly enriched in astrocytes (Mukai et al., 1986; Zhang et al., 2014), is expressed at low levels in patients with AD and Parkinson’s disease and is correlated with impaired glucose metabolism (Peng et al., 2024; Wang et al., 2021). Notably, aldolase can regulate AMP-activated protein kinase (AMPK) by sensing FBP levels (Zhang et al., 2017). For example, in oligodendrocyte precursor cells under low-glucose conditions, ALDOC acetylation suppresses AMPK activity, thereby promoting myelination (Sun et al., 2025). The AMPK/mTOR/HIF-1α axis is central to metabolic reprogramming (Baik et al., 2019). AMPK acts as an energy sensor; its activation under glucose deprivation can occur via an aldolase-FBP interaction that modulates the AMPK-axin complex (Lin and Hardie, 2018). AMPK subsequently suppresses mechanistic target of rapamycin (mTOR) complex 1 (Inoki et al., 2003), while hypoxia-inducible factor-1α (HIF-1α), a transcriptional regulator of glycolysis, is also modulated by mTOR signaling (Porel et al., 2025). In AD, Aβ exposure triggers microglial metabolic shifts that depend on the mTOR-HIF-1α axis (Baik et al., 2019), and astrocytic HIF-1α-mediated glycolysis is impaired (de Sánchez Muniain et al., 2025).

Magnesium (Mg) is an essential cofactor for over 300 enzymes, including those involved in ATP metabolism (Xiong et al., 2025). Mg deficiency is associated with AD, and supplementation improves cognitive function and Aβ pathology (Xiong et al., 2025; Salinas et al., 2023). However, it remains unclear whether Mg promotes astrocytic ALDOC expression and regulates metabolic reprogramming via the AMPK/mTOR/HIF-1α axis in AD.

In this study, we observed significant downregulation of ALDOC in AD astrocytes, accompanied by alterations in the AMPK/mTOR/HIF-1α axis and reduced expression of the glycolytic enzymes LDHA and PKM2. These findings led us to investigate the molecular mechanisms by which ALDOC regulates astrocyte metabolism and its functional consequences for neuronal viability.

## Materials and methods

### Animals

Male C57BL/6 wild-type mice (WT, 6 months old, 20–25 g) and male APP/PS1 mice (6 months old, 20–25 g) were obtained from Fuhuakang Biotechnology Co., Ltd. (Beijing, China). All the animals were housed under standardized conditions with a 12 h light/dark cycle, an ambient temperature of 22 ± 3 °C, and a relative humidity of 60% ± 5%. The mice were provided ad libitum access to food and water throughout the experimental period. At the end of the experimental period, the temporal cortex and hippocampal CA1 region were dissected for subsequent molecular analyses. All procedures were conducted in strict compliance with the Guidelines for the Management of Laboratory Animals issued by the Ministry of Science and Technology of the People’s Republic of China and were approved by the Institutional Animal Care and Use Committee (IACUC) of the Second Hospital of Hebei Medical University (approval number: 2025-AE404). Efforts were made to minimize animal suffering and to adhere to the principles of the 3Rs (replacement, reduction, and refinement) in animal research.

### Preparation of oligo-Aβ

Aβ1–42 peptides (synthesized by China MeilunBio Co., Ltd.) were dissolved in 1,1,1,3,3,3-hexafluoro-2-propanol (HFIP; Sigma–Aldrich) to a final concentration of 1 mM. HFIP was evaporated under vacuum using a SpeedVac Concentrator (Savant Instruments), and the dried peptides were stored at −80 °C. The Aβ1-42 peptide was dissolved to 1 mM in anhydrous dimethyl sulfoxide (anhydrous DMSO; Sigma–Aldrich). Oligo-Aβ (oAβ) was obtained by diluting the peptide to 5 μM in DMEM. The resulting solutions were then incubated overnight at 4 °C (Baik et al., 2019).

### Cell culture and treatment

Human astrocyte SVGp12 and human neuroblastoma SH-SY5Y cells were obtained from Shanghai Zhong Qiao Xin Zhou Biotechnology Co., Ltd. (Shanghai, China). Cells were cultured in DMEM (Gibco, USA) supplemented with 100 U/mL penicillin, 100 μg/mL streptomycin (Pen-Strep, Gibco, USA), and 10% fetal bovine serum (FBS, Gibco, USA). The cells were maintained in a humidified incubator at 37 °C with 5% CO_2_. When cells reached approximately 80–90% confluence, they were digested with 0.25% trypsin and passaged at a 1:3–1:4 ratio. Dishes were placed in a 37 °C, 5% CO_2_ incubator and then passaged approximately every 3 days. SVGp12 cells were treated with 5 μM oAβ for 24 h to prepare AD cellular models. According to the experimental design protocol, 1 mM, 4 mM, and 8 mM magnesium sulfate (MCE, HY-Y1267D, USA) were applied to SVGp12 cells at different stages for 24 h (Xiong et al., 2022; Xiong et al., 2025).

### Cell coculture

To assess the energetic support effect of SVGp12 cells on SH-SY5Y cells, SH-SY5Y cells (upper chamber) and SVGp12 cells (lower chamber) were cocultured using a Transwell system (0.4 μm pore size, 6-well plate). SH-SY5Y cells were seeded at a density of 2 × 10^4^ cells/well in the upper chamber of the Transwell, and SVGp12 cells were seeded at 2 × 10^5^ cells/well in the lower chamber of the Transwell. After the cells adhered and grew to 70% confluence, oAβ was added to the lower chamber for 24 h. Blank plasmid and ALDOC overexpression plasmid were selectively added to the lower chamber according to the experimental design. To inhibit the expression of MCT2 protein in SH-SY5Y cells, the cells were seeded in a six-well plate, grown to 80–90% confluence, treated with 3 μM AR-C155858 (MedchemExpress, HY-13248, USA) for 2 h, and then digested with 0.25% trypsin, seeded in the upper chamber of a Transwell plate, and cocultured with the SVGp12 cells in the lower chamber. The dose of AR-C155858 was determined according to previous literature (Guan et al., 2018, 2019).

### Cell viability assay

Relative cell viability was measured via a cell counting kit (CCK-8) assay. The cells to be tested were seeded in a 96-well plate at 2,000 cells per well in a cell incubator. The cells were incubated with 10 μL of CCK-8 reagent (Zeta Life, USA) per well for 3 h at 37 °C in accordance with the manufacturer’s instructions. The absorbance was measured at 450 nm using a multimode microplate reader. Reproducibility and precision were ensured by preparing at least six batches of each experimental sample.

### Construction of overexpression plasmids and small interfering RNAs (siRNAs)

We prepared pcDNA3.1(+)-ALDOC (Gene ID: 230, NM_005165.3), pcDNA3.1(+)-HIF-1α (Gene ID: 3091, NM_001530.4), and pcDNA3.1(+)-nc lentiviral plasmids provided by Suzhou Gene Pharma Co., Ltd. (Suzhou, China). pcDNA3.1(+)-nc was used as a negative control. In brief, approximately 24 h before transfection, exponentially growing cells were trypsinized, resuspended in complete culture medium without antibiotics, and seeded in 24-well plates at a density of 5 × 10^4^ to 1 × 10^5^ cells per well. The cells were then incubated overnight at 37 °C with 5% CO₂ to reach 70–90% confluency at the time of transfection. A total of 0.5–1.0 μg of plasmid DNA was diluted in 50 μL of serum-free Opti-MEM. Simultaneously, an appropriate amount of Lipofectamine 3000 reagent was diluted in an equal volume of Opti-MEM. After incubation at room temperature for 5 min, the two diluted solutions were combined, gently mixed by pipetting, and further incubated for 15–20 min at room temperature to form DNA-transfection reagent complexes. The complexes were then added dropwise to the cell culture wells, and the plate was gently rocked to ensure even distribution. At 4–6 h post-transfection, the medium containing the complexes was replaced with fresh complete medium to mitigate cytotoxicity. Cells were harvested 24–72 h after transfection for downstream analyses to evaluate the transfection efficiency and target gene expression.

siRNAs were transfected in approximately the same way as the plasmids above. We also prepared four siRNA sequences targeting ALDOC and HIF-1α, along with a negative control siRNA sequence, as shown in Table 1; these sequences were all from Suzhou Gene Pharma Co., Ltd. (Suzhou, China). SiRNA (si-nc) was used as a negative control.

**Table 1:** siRNA sequences.

Strand type	siRNA ID	Sequences
si-ALDOC-613	Sense strand	5′-GCGUACACCCUCUGCACUUTT-3′
	Antisense strand	5′-AAGUGCAGAGGGUGUACGCTT-3′
si-ALDOC-739	Sense strand	5′-CCUCAAACGUUGUCAGUAUTT-3′
	Antisense strand	5′-AUACUGACAACGUUUGAGGTT-3′
si-ALDOC-790	Sense strand	5′-GGCCCUGAGUGACCAUCAUTT-3′
	Antisense strand	5′-AUGAUGGUCACUCAGGGCCTT-3′
si-ALDOC-930	Sense strand	5′-GCGAAGAAGAGGCAUCAUUTT-3′
	Antisense strand	5′-AAUGAUGCCUCUUCUUCGCTT-3′
si-HIF1α-934	Sense strand	5′-CGAGGAAGAACUAUGAACATT-3′
	Antisense strand	5′-UGUUCAUAGUUCUUCCUCGTT-3′
si-HIF1α-1168	Sense strand	5′-GAUGAAAGAAUUACCGAAUTT-3′
	Antisense strand	5′-AUUCGGUAAUUCUUUCAUCTT-3′
si-HIF1α-1553	Sense strand	5′-GUAGCCUCUUUGACAAACUTT-3′
	Antisense strand	5′-AGUUUGUCAAAGAGGCUACTT-3′
si-HIF1α-2090	Sense strand	5′-CUCCCUAUAUCCCAAUGGATT-3′
	Antisense strand	5′-UCCAUUGGGAUAUAGGGAGTT-3′
Negative control	Sense strand	5′-UUCUCCGAACGUGUCACGUTT-3′
	Antisense strand	5′-ACGUGACACGUUCGGAGAATT-3′

### Sample protein extraction and quantification

Samples from different groups were lysed using RIPA buffer (Solarbio, R0020, China) supplemented with phosphatase and protease inhibitors (GenDEPOT, USA). The lysates were sonicated and centrifuged at 12,000 rpm for 20 min at 4 °C, after which the supernatants were collected. A BCA protein assay reagent kit (Novagen, Madison, WI, USA) was used to determine the protein concentrations.

### Western blot analysis

Equal amounts of protein (50 μg) were separated by SDS–PAGE, transferred to PVDF membranes (Roche, USA), and blocked with 5% nonfat milk. Membranes were incubated overnight at 4 °C with the following primary antibodies: anti-Aldolase C (Abcam, ab87122, 1 μg/mL); anti-AMPK alpha 1 [Y365] (Abcam, ab32047, 1:1,000); anti-AMPK alpha 2 (phospho T172) [4F4] (Abcam, ab314032, 1:1,000); anti-mTOR (Cell Signaling Technology, #2972, 1:1,000); anti-phospho-mTOR (Cell Signaling Technology, #5536, 1:1,000); anti-HIF-1α (D1S7W) (Cell Signaling Technology, #36169, 1:1,000); anti-PKM2 (D78A4) (Cell Signaling Technology, #4053, 1:1,000); anti-LDHA (C4B5) (Cell Signaling Technology, #3582, 1:1,000); anti-MCT2/SLC16A7 (Proteintech, 20355-1-AP,1:1,000); anti-beta Tubulin 3/Tuj1(Genetex, GTX130245, 1:1,000); anti-GLT-1 (Proteintech, 67083-1-Ig, 1:1,000); and anti-beta Actin (Genetex, GTX109639, 1:10,000). After being washed with TBST, the membranes were incubated with HRP-conjugated secondary antibodies for 1 h at 37 °C. The relative density of the bands was analyzed with an Odyssey infrared scanner (LI-COR Bioscience, USA). At least six independent experiments were performed for each batch of samples (*n* = 6). Band intensities were quantified using ImageJ software, and the expression of each target protein was normalized to its corresponding internal reference band. The normalized data from the treatment groups are expressed as fold changes relative to those of the control group.

### Immunofluorescence

Anesthetized mice were transcardially perfused with PBS followed by 4% paraformaldehyde. Brains were obtained, and the brain tissues were fixed with 4% paraformaldehyde for 24 h, followed by gradient dehydration using 30, 25, and 20% sucrose solutions for 48 h. Coronal sections (15 μm thick) were prepared using a freezing cryotome (Thermo Fisher Scientific, USA). The sections were blocked with PBS containing 0.3% Triton X-100 and 10% normal goat serum for 1 h at room temperature, then incubated with primary antibodies overnight at 4 °C. The following primary antibodies were used: *β*-Amyloid (D54D2) (Cell Signaling Technology, #8243, 1:800), anti-Aldolase C (Abcam, ab87122, 5 μg/mL), anti-HIF-1α (D1S7W) (Cell Signaling Technology, #36169, 1:100), anti-LDHA (C4B5) (Cell Signaling Technology, #3582, 1:500), and anti-Glial Fibrillary Acidic Protein Antibody (Sigma–Aldrich, MAB360, 1:500). On the following day, the sections were rinsed and incubated with appropriate fluorescent secondary antibodies in humidified chambers at 37 °C for 1 h. At least three independent experiments were performed for each batch of samples. Coronal hippocampal sections were selected for analysis, and the data were quantified using ImageJ software. Fluorescence intensity was quantified in whole microscopic fields. For APP/PS1 mice, only fields containing at least one Aβ plaque were analyzed; for wild-type mice, fields were randomly selected from the same brain regions (cortex/hippocampus). For each mouse, 3 fields per region were measured, and the mean intensity per field was used for comparison.

For cellular immunofluorescence, cells were first plated in 24-well plates and, after adhering and differentiating, fixed with 4% paraformaldehyde, following steps consistent with those of the tissue immunofluorescence assay. The following primary antibodies were used: anti-Aldolase C (Abcam, ab87122, 5 μg/mL), anti-HIF-1α (D1S7W) (Cell Signaling Technology, #36169, 1:100), anti-LDHA (C4B5) (Cell Signaling Technology, #3582, 1:500), anti-Glial Fibrillary Acidic Protein Antibody (Sigma–Aldrich, MAB360, 1:500), anti-MCT2/SLC16A7 (Proteintech, 20355-1-AP, 1:200), and anti-FOX3/NeuN [1B7] (Arigo, ARG52283, 1:300).

For immunofluorescence quantification, images were acquired under the same microscope settings. Quantitative analysis was performed using ImageJ software. Background intensity values were measured from cell-free regions of each image and subtracted. To ensure objectivity, a uniform threshold for positive signal detection was automatically determined using a “moment” algorithm applied to all control group images, then applied to all treatment group images. The integrated fluorescence density (IFD) was calculated as follows: (mean fluorescence intensity—background) × positive area. To account for changes in cell density, the number of nuclei was counted using DAPI staining, and the IFD was normalized to the number of cells in each field.

Colocalization of ALDOC and GFAP in the hippocampus and cerebral cortex was quantified using the Coloc 2 plugin in FIJI (ImageJ). After automatic thresholding to exclude background noise, Pearson’s R and thresholded Manders’ coefficients (tM1, tM2) were calculated.

### Mitochondrial membrane potential detection

Mitochondrial membrane potential was determined using a JC-1 probe (M8650, Solarbio). Astrocytes were pre-cultured in 6-well plates, and 1 mL of fresh medium was added to each well to replace the old medium. Then, 1 mL of JC-1 solution was added to each well, and the cells were incubated for 20 min at 37 °C. After the medium was removed, 2 mL of fresh medium was added to each cell. Mitochondrial membrane potential was determined using a fluorescence microscope at Ex/Em = 490/530 nm.

### Polymerase chain reaction (qRT-PCR)

qRT-PCR was used to analyze mRNA expression levels of different groups (*n* = 6 per group). Total RNA from the cells was isolated using TRIzol reagent (Tiangen Biotechnology) and reverse-transcribed into cDNA using a PrimeScript™ RT reagent kit with gDNA Eraser (TaKaRa). We carried out amplification and detection using an ABI 7500 Real-Time PCR system (Applied Biosystems) with SYBR^®^ Premix ExTaq™ II (Tli RNaseH Plus) and ROX plus (TaKaRa). The results for each sample were determined at least six times. The relative abundance of mRNA from the prospective target genes was calculated via the 2^−ΔΔCT^ method after normalization to ACTB RNA. All primers (DNA sequence 5′ -3′) were pooled (Table 2).

**Table 2:** Primer sequences for qRT-PCR.

Gene	Orientation	Primer sequence
ALDOC	Forward	5′-CGC ACAGGGAGCTGTCAC-3′
	Reverse	5′-CCTCTGTTTTCCACCCCA-3′
HIF-1α	Forward	5′-TGGGGCAGTCAATGGATGAG-3′
	Reverse	5′-AAATAGACTGCTGAGCCACC-3′
PKM2	Forward	5′-TCACCCTGGACAACGCTTAC-3′
	Reverse	5′-AGTCAGCGCCTTTCTCCTTC-3′
LDHA	Forward	5′-AGTAAGTCCTCAGGCGGCTA-3′
	Reverse	5′-TGAGGGTTGCCATCTTGGAC-3′
ACTB (human)	Forward	5′-CACCATTGGCAATGAGCGGTTC-3′
	Reverse	5′-AGGTCTTTGCGGATGTCCACGT-3′
ACTB (mouse)	Forward	5′-CCCATCTATGAGGGTTACGC-3′
	Reverse	5′-TTTAATGTCACGCACGATTTC-3′

### ATP measurement

Intracellular ATP levels were determined using an ATP Assay Kit (Beyotime Biotechnology, China) according to the manufacturer’s instructions. In brief, the ATP detection working buffer (100 μL) was gently mixed with the substrate, and the luminescence was subsequently measured using a microplate reader (BioTek, China) at various time points. All the experimental samples were subjected to six independent experiments.

### Extracellular acidification rate assay

An extracellular acidification rate (ECAR) fluorescence test box (Elabscience, E-BC-F069, China) was used to measure ECAR according to the manufacturer’s instructions. The cells were inoculated into 96-well plates at a density of 2 × 10^5^ cells/ml. In accordance with the experimental design, the corresponding drugs were added to stimulate the cells. After incubation at 37 °C in the dark for 30 min, 100 μL of working solution was added to each well. A fluorescence microplate reader was used to determine the fluorescence value of each well at an excitation wavelength of 490 nm and an emission wavelength of 535 nm. The ECAR rate was calculated from the fluorescence time curve. Reproducibility and precision were ensured by preparing at least six batches of each experimental sample.

### L-lactate assay

L-lactate contents were measured using an L-lactate assay kit (Abcam, ab65330) according to the manufacturer’s instructions. Cells (10^6^ cells) were washed twice with PBS, homogenized with lactate assay buffer, and centrifuged for 5 min at 4 °C and 12,000 rpm. The resulting supernatants were collected. The reaction reagents were prepared as instructed and mixed with 50 μL of the samples, which were then incubated at room temperature in the dark for 30 min, after which the OD was measured at 570 nm. Six independent experiments were conducted for each sample (*n* = 6).

### Statistical analysis

All the experiments were performed with at least three independent biological replicates (*n* ≥ 3). A biological replicate was defined as cells cultured/treated or animals processed independently on different days. The results were consistent across all the independent replicates. Statistical analysis was performed using SPSS 27.0, and the data are presented as means ± standard errors of the mean (SEM). Unpaired Student’s t tests were used for comparisons between two groups, and one-way analysis of variance (ANOVA) was used for multiple samples. A *p-*value of < 0.05 was considered to indicate statistical significance. All the statistical graphs were generated using GraphPad Prism 8.0 software (San Diego, USA).

## Results

### Downregulation of ALDOC is accompanied by decreased expression of key glycolytic proteins in APP/PS1 mice

**Figure 1:**
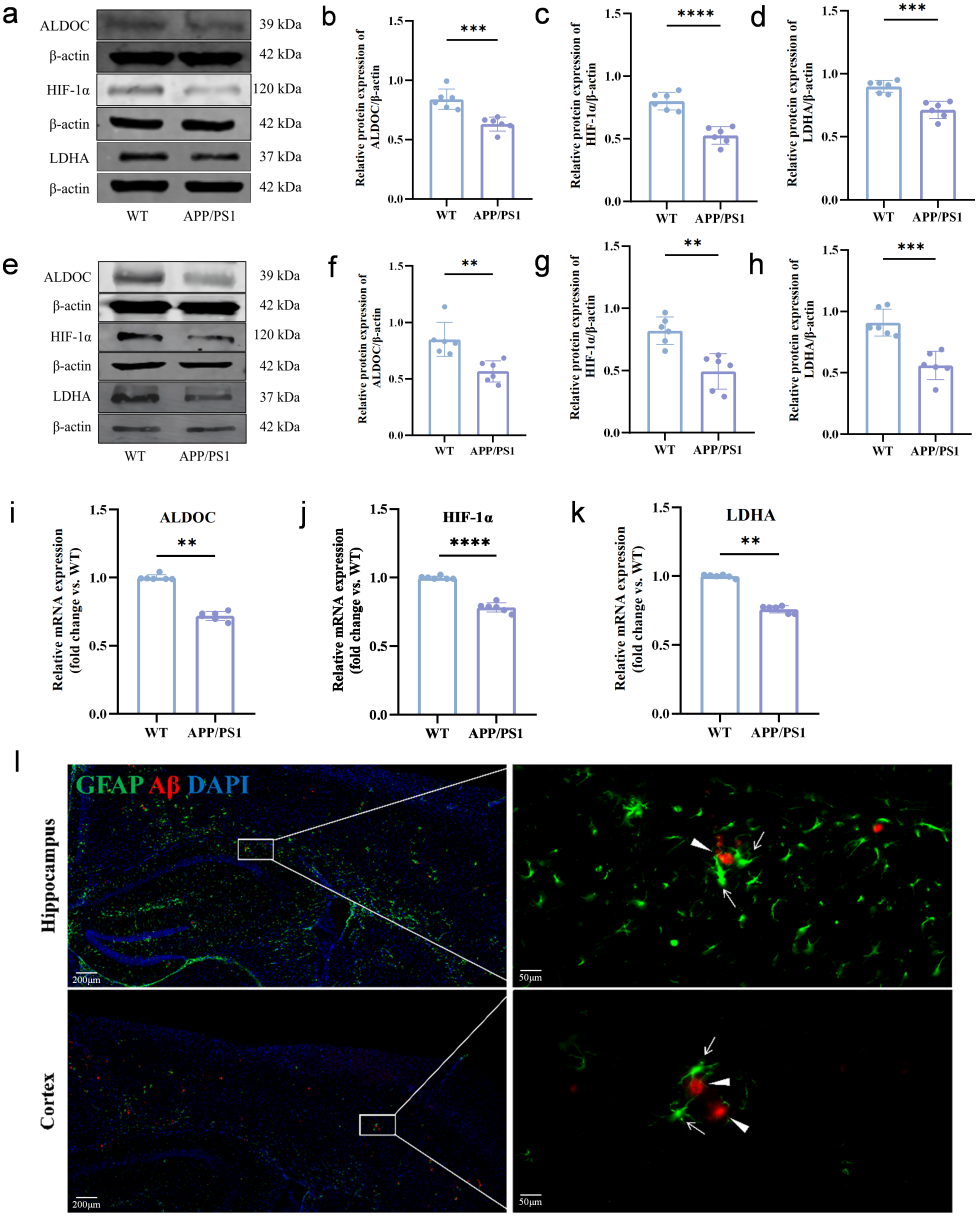
APP/PS1 mice showed decreased expression of ALDOC and key glycolytic proteins in reactive astrocytes compared with age-matched wild-type mice. **(a)** Western blot image of ALDOC, HIF-1α, and LDHA in the hippocampus of C57BL/6 J wild-type (WT) mice and APP/PS1 transgenic mice. **(b–d)** Quantitative analysis of protein expression of ALDOC, HIF-1α, and LDHA in the hippocampus (*n* = 6). **(e)** Western blot image of ALDOC, HIF-1α, and LDHA in the cerebral cortex of C57BL/6 J wild-type mice and APP/PS1 transgenic mice. **(f–h)** Quantitative analysis of protein expression of ALDOC, HIF-1α, and LDHA in the cerebral cortex (*n* = 6). **(i–k)** Relative mRNA levels of ALDOC, HIF-1α, and LDHA in the hippocampus of C57BL/6 J wild-type mice and APP/PS1 transgenic mice (*n* = 6). **(l)** APP/PS1 mouse brain tissue showed a large amount of Aβ amyloid deposition in the hippocampus and cortex (white triangles), accompanied by surrounding reactive astrocytes (white arrows). **(a–k)** All experiments were performed independently using samples derived from at least three different batches (*n* ≥ 3 per group). **p < 0.01, ***p < 0.001, ****p < 0.0001, ns (non-significant). Scale bars are indicated directly on the image.

We first examined ALDOC expression in the hippocampus and cerebral cortex of APP/PS1 transgenic mice. Western blot analysis revealed significant decreases in ALDOC protein levels in both the hippocampus (*p* < 0.001; Figures 1a,b) and cerebral cortex (*p* < 0.01; Figures 1e,f) compared with those in wild-type (WT) mice. Consistently, the protein levels of HIF-1α and LDHA were also significantly reduced in both brain regions (hippocampus: HIF-1α, *p* < 0.0001, LDHA, *p* < 0.001; Figures 1a,c,d; cortex: HIF-1α, *p* < 0.01, LDHA, *p* < 0.001; Figures 1e,g,h). Similar reductions in ALDOC, HIF-1α, and LDHA mRNA expression were observed in the hippocampal tissues (Figures 1i–k).

Immunofluorescence (IF) staining confirmed the astrocytic localization of ALDOC, as shown by its high colocalization with the astrocytic marker GFAP (Pearson’s *R* = 0.77; Manders’ colocalization coefficients above autothreshold: tM1 = 0.356, tM2 = 0.964; Supplementary Figures S1a,j). Conversely, ALDOC exhibited minimal colocalization with the neuronal marker NeuN (Pearson’s *R* = 0.11; tM1 = 0.122, tM2 = 0.350; Supplementary Figures S1i,k), supporting the specificity of ALDOC expression in astrocytes. In APP/PS1 mice, Aβ deposits were surrounded by reactive astrocytes with hypertrophic cell bodies and elongated processes (Figure 1l). Quantification of IF signals revealed that the relative fluorescence intensities of ALDOC, HIF-1α, and LDHA were significantly reduced around Aβ deposits in both the hippocampus (*p* < 0.01 for each; Supplementary Figures S1a–d) and cerebral cortex (*p* < 0.01 for each; Supplementary Figures S1e–h) compared with those in WT controls.

### oAβ treatment reduces ALDOC expression and impairs glycolysis in astrocytes

**Figure 2:**
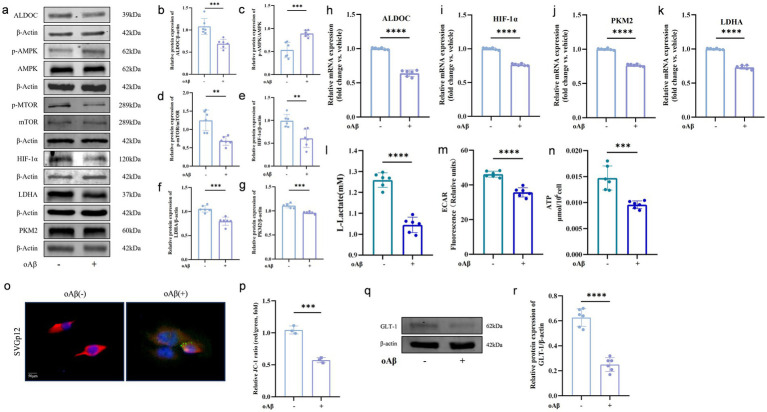
Metabolic alterations in SVGp12 astrocytes following oAβ exposure. **(a)** Western blot image of ALDOC, pAMPK, AMPK, pMTOR, mTOR, HIF-1α, LDHA, and PKM2 in non-intervened SVGp12 cells and oAβ-intervened SVGp12 cells. **(b–g)** Quantitative analysis of protein expression of ALDOC, pAMPK, AMPK, pMTOR, mTOR, HIF-1α, LDHA, and PKM2 in **(a)** (*n* = 6). **(h–k)** Relative mRNA expression levels of ALDOC, HIF-1α, LDHA, and PKM2 in non-intervened SVGp12 cells versus oAβ-intervened SVGp12 cells (*n* = 6). **(l–n)** L-lactate level, ECAR level, and ATP content in non-intervened SVGp12 cells versus oAβ-intervened SVGp12 cells (*n* = 6). **(o)** Effect of oAβ treatment on JC-1 staining in SVGp12 cells and the corresponding statistical graph of the red/green fluorescence ratio **(p)**. **(q)** Western blot image of GLT-1 in none-intervened SVGp12 cells and oAβ-intervened SVGp12 cells, and quantitative analysis of protein expression of GLT-1. **(a–r)** All experiments were performed independently using samples derived from at least three different batches (*n* ≥ 3 per group). *p* < 0.01, *p* < 0.001, *p* < 0.001, ns (non-significant). Scale bars are indicated directly on the image.

To establish an AD cellular model, the human astrocyte cell line SVGp12 was treated with oligomeric Aβ (oAβ) for 24 h (oAβ + SVGp12 group), with untreated cells serving as controls (Zhang et al., 2021). Western blot analysis revealed that oAβ treatment significantly reduced ALDOC protein expression (*p* < 0.001; Figures 2a,b). This reduction was accompanied by an increased p-AMPK/AMPK ratio (*p* < 0.001; Figures 2a,c) and a decreased p-mTOR/mTOR ratio (*p* < 0.01; Figures 2a,d), indicating altered AMPK/mTOR signaling. The protein levels of HIF-1α (*p* < 0.01; Figures 2a,e), as well as the glycolytic enzymes LDHA (*p* < 0.001; Figures 2a,f) and PKM2 (*p* < 0.001; Figures 2a,g), were also decreased following oAβ treatment. Quantitative real-time PCR confirmed the reduced mRNA levels of ALDOC (*p* < 0.0001; Figure 2h), HIF-1α (*p* < 0.0001; Figure 2i), PKM2 (*p* < 0.0001; Figure 2j), and LDHA (*p* < 0.0001; Figure 2k) in oAβ-treated cells. Immunofluorescence analysis further revealed decreased fluorescence intensity of ALDOC (*p* < 0.05; Supplementary Figures S2a,b), HIF-1α (*p* < 0.05; Supplementary Figures S2a,c), and LDHA (*p* < 0.05; Supplementary Figures S2a,d) in oAβ-treated cells compared with control cells.

To assess glycolytic function, we measured lactate production, the ECAR, and the cellular ATP content. Both lactate levels (*p* < 0.0001; Figure 2l) and the ECAR (*p* < 0.0001; Figure 2m) were significantly lower in the oAβ + SVGp12 group than in the control group. Consistent with impaired glycolysis, cellular ATP content was also decreased (*p* < 0.001; Figure 2n). Collectively, these results indicate that oAβ treatment reduces ALDOC expression and is associated with impaired glycolytic capacity in SVGp12 cells.

Additionally, JC-1 staining was performed to assess changes in mitochondrial membrane potential. The relative JC-1 red/green fluorescence ratio was significantly decreased in oAβ-treated SVGp12 cells compared with controls (*p* < 0.001; Figures 2o,p). Moreover, western blot analysis showed that oAβ treatment markedly downregulated the expression of glutamate transporter-1 (GLT-1), an astrocytic glutamate transporter, in SVGp12 cells (*p* < 0.0001; Figures 2q,r). This result further indicates that astrocytes exhibit functional impairment following oAβ intervention.

### ALDOC regulates glycolytic capacity in oAβ-treated astrocytes through HIF-1α

**Figure 3:**
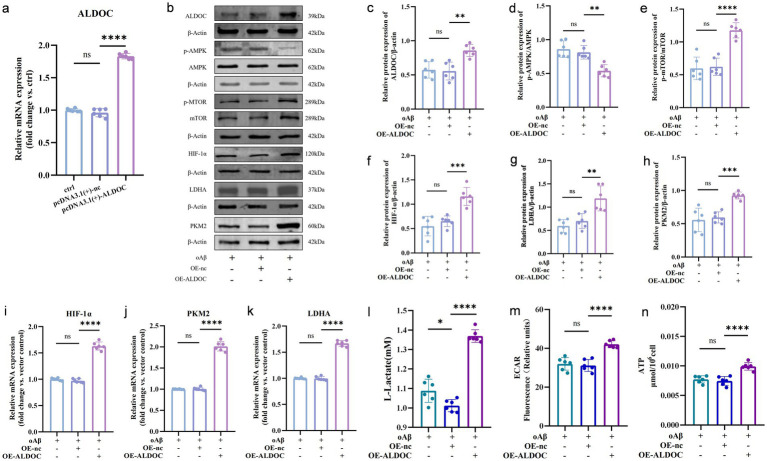
Modulating ALDOC expression in SVGp12 cells, followed by oAβ intervention, consequently altered the expression of the AMPK-mTOR-HIF1α pathway and affected glycolytic levels. **(a)** The overexpression effect of the ALDOC overexpression plasmid (*n* = 6 independent biological replicates). **(b)** Western blot image of ALDOC, pAMPK, AMPK, pMTOR, mTOR, HIF-1α, LDHA, and PKM2 in oAβ + SVGp12 cells with ALDOC gene overexpressed, or none overexpressed. **(c–h)** Quantitative protein analysis of ALDOC, pAMPK, AMPK, pMTOR, mTOR, HIF-1α, LDHA, and PKM2 in **(b)** (*n* = 6). **(i–k)** Relative mRNA expression levels of HIF-1α, LDHA, and PKM2 in oAβ + SVGp12 cells following ALDOC overexpression (*n* = 6). **(l–n)** L-lactate level, ECAR level, ATP content in oAβ + SVGp12 cells following ALDOC overexpression (*n* = 6). **(a–n)** All experiments were performed independently using samples from at least six different batches (*n* = 6 per group). *p < 0.05, **p < 0.01, ***p < 0.001, ****p < 0.0001, ns (non-significant).

To further investigate the functional role of ALDOC in AD, we constructed an ALDOC overexpression plasmid (OE-ALDOC) and a corresponding negative control plasmid (OE-nc) and transfected them into SVGp12 cells, followed by oAβ intervention. Increased ALDOC expression was confirmed at both the mRNA (*p* < 0.0001; Figure 3a) and protein levels (*p* < 0.01; Figures 3b,c) in OE-ALDOC-transfected cells. The cells were divided into three experimental groups: oAβ + SVGp12, oAβ + SVGp12 + OE-nc, and oAβ + SVGp12 + OE-ALDOC. Western blot analysis revealed that compared with the OE-nc group, the ALDOC-overexpressing group had lower p-AMPK/AMPK ratios (*p* < 0.01; Figures 3b,d), higher p-mTOR/mTOR ratios (*p* < 0.0001; Figures 3b,e), and higher protein levels of HIF-1α (*p* < 0.001; Figures 3b,f), LDHA (*p* < 0.01; Figures 3b,g), and PKM2 (*p* < 0.001; Figures 3b,h). Consistent with these findings, ALDOC overexpression also increased the mRNA levels of HIF-1α (*p* < 0.0001; Figure 3i), PKM2 (*p* < 0.0001; Figure 3j), and LDHA (*p* < 0.0001; Figure 3k). Functionally, ALDOC overexpression in oAβ-treated SVGp12 cells partially rescued the impaired glycolytic phenotype, as evidenced by elevated lactate production (*p* < 0.0001; Figure 3l) and an increased ECAR (*p* < 0.0001; Figure 3m). The cellular ATP content was also markedly restored (*p* < 0.0001; Figure 3n).

To further investigate the role of ALDOC, we designed a siRNA to knock down ALDOC expression in an AD cellular model. After screening a set of prepared siRNAs, we identified si-ALDOC-739 (hereafter abbreviated as si-ALDOC) as the most effective construct, resulting in a significant reduction in ALDOC mRNA expression (*p* < 0.001; Supplementary Figure S3a). In subsequent experiments, the cells were divided into three treatment groups: oAβ + SVGp12, oAβ + SVGp12 + si-nc (negative control), and oAβ + SVGp12 + si-ALDOC. Western blot analysis confirmed that ALDOC knockdown reduced ALDOC protein levels compared with those in the si-nc group (*p* < 0.01; Supplementary Figures S3b,c). In response to oAβ treatment, ALDOC knockdown was associated with an increased p-AMPK/AMPK ratio (*p* < 0.001; Supplementary Figures S3b,d), a decreased p-mTOR/mTOR ratio (*p* < 0.0001; Supplementary Figures S3b,e), and reduced protein levels of HIF-1α (*p* < 0.01; Supplementary Figures S3b,f), LDHA (*p* < 0.001; Supplementary Figures S3b,g), and PKM2 (*p* < 0.01; Supplementary Figures S3b,h). Quantitative PCR analysis confirmed that ALDOC knockdown also reduced the mRNA levels of HIF-1α (*p* < 0.05; Supplementary Figures S3i), PKM2 (*p* < 0.05; Supplementary Figures S3j), and LDHA (*p* < 0.0001; Supplementary Figure S3k), which is consistent with the western blot results. Functionally, ALDOC knockdown further impaired glycolysis in oAβ-treated SVGp12 cells, as evidenced by decreased lactate production (*p* < 0.0001; Supplementary Figure S3l), decreased ECAR (*p* < 0.01; Supplementary Figure S3m), and reduced ATP content (*p* < 0.01; Supplementary Figure S3n).

**Figure 4:**
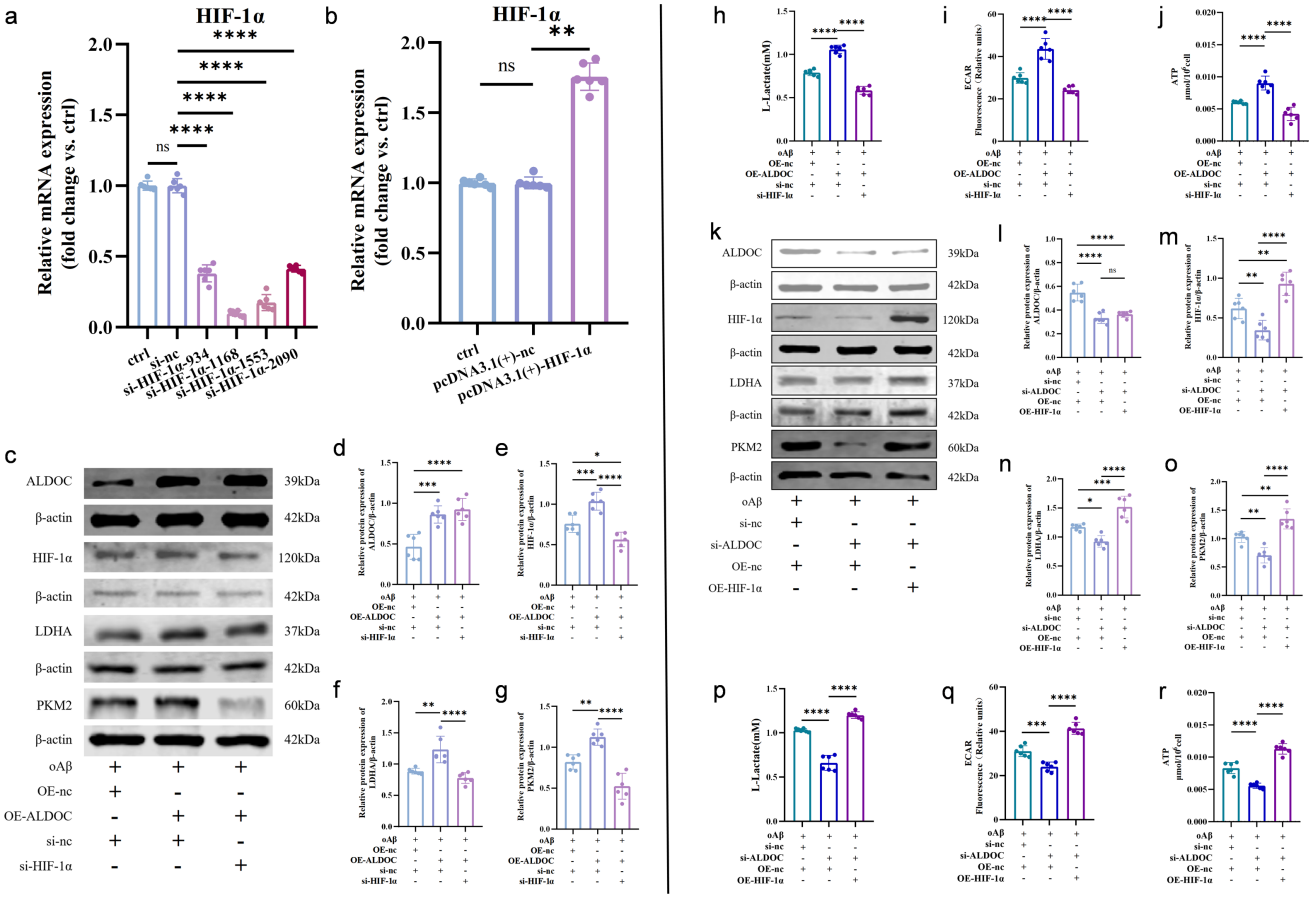
ALDOC is associated with metabolic changes in oAβ-exposed astrocytes, possibly involving the AMPK/mTOR/HIF-1α pathway. **(a)** Knockdown efficiency of the HIF-1α gene by different siRNAs (*n* = 6). **(b)** The overexpression effect of the ALDOC overexpression plasmid (*n* = 6). **(c)** Western blot image of ALDOC, HIF-1α, LDHA, and PKM2 in oAβ-induced SVGp12 cells following ALDOC overexpression with or without HIF-1α knockdown. **(d–g)** Quantitative protein analysis of ALDOC, HIF-1α, LDHA, and PKM2 in **(c)** (*n* = 6). **(h–j)** L-lactate level, ECAR level, and ATP content in oAβ induced SVGp12 cells following ALDOC overexpression with or without HIF-1α knockdown (*n* = 6). **(k)** Western blot image of ALDOC, HIF-1α, LDHA, and PKM2 in oAβ-induced SVGp12 cells following ALDOC knockdown with or without HIF-1α overexpression. **(l–o)** Quantitative protein analysis of ALDOC, HIF-1α, LDHA, and PKM2 in (k) (*n* = 6). **(p–r)** L-lactate level, ECAR level, ATP content in oAβ induced SVGp12 cells following ALDOC knockdown with or without HIF-1α overexpression (*n* = 6). **(a–r)** All experiments were performed independently using samples from at least six different batches (*n* = 6 per group). *p < 0.05, **p < 0.01, ***p < 0.001, ****p < 0.0001, ns (non-significant).

To further investigate the mechanism by which ALDOC regulates glycolytic capacity in SVGp12 cells, we performed rescue experiments in which HIF-1α, identified as a potential downstream effector, was targeted based on previous observations. We first selected an efficient siRNA, si-HIF-1α-1168 (hereafter, si-HIF-1α), which significantly reduced HIF-1α expression (*p* < 0.0001; Figure 4a), and then validated a HIF-1α overexpression plasmid (OE-HIF-1α) that robustly increased its expression (*p* < 0.01; Figure 4b).

In the first set of experiments, the cells were divided into three groups: the control group (oAβ + SVGp12 + si-nc + OE-nc), the ALDOC overexpression group (oAβ + SVGp12 + si-nc + OE-ALDOC), and the rescue group (oAβ + SVGp12 + si-HIF-1α + OE-ALDOC). As expected, compared with the control group, ALDOC was successfully overexpressed (*p* < 0.001; Figures 4c,d) and HIF-1α was knocked down (*p* < 0.05; Figures 4c,e) in the rescue group. Notably, despite resulting in overexpression of ALDOC, HIF-1α knockdown suppressed the expression of the glycolytic enzymes LDHA (*p* < 0.0001; Figures 4c,f) and PKM2 (*p* < 0.0001; Figures 4c,g). Functionally, this effect was accompanied by reduced lactate production (*p* < 0.0001; Figure 4h), ECAR (*p* < 0.0001; Figure 4i), and ATP content (*p* < 0.0001; Figure 4j), suggesting that HIF-1α is required for the full glycolytic effect of ALDOC overexpression. In a complementary approach, we overexpressed HIF-1α under ALDOC knockdown conditions (Figures 4l,m). Compared with the ALDOC knockdown group (oAβ + SVGp12 + si-ALDOC + OE-nc), HIF-1α overexpression (oAβ + SVGp12 + si-ALDOC + OE-HIF-1α) significantly increased the expression of LDHA (*p* < 0.0001; Figures 4k,n) and PKM2 (*p* < 0.0001; Figures 4k,o), lactate production (*p* < 0.0001; Figure 4p), the ECAR (*p* < 0.0001; Figure 4q), and ATP levels (*p* < 0.0001; Figure 4r). These results are consistent with those of previous reports on the role of HIF-1α in regulating glycolytic enzymes (Chen et al., 2018; Tyszka-Czochara et al., 2018).

Taken together, the findings of these rescue experiments indicate that HIF-1α acts as a downstream mediator of the regulation of ALDOC-associated glycolysis in this cellular model. The coordinated changes in p-AMPK, p-mTOR, and HIF-1α following alterations in ALDOC expression further support the involvement of the AMPK/mTOR/HIF-1α signaling axis in ALDOC-driven glycolytic modulation.

### ALDOC overexpression in astrocytes improves neuronal energy support in a coculture model

**Figure 5:**
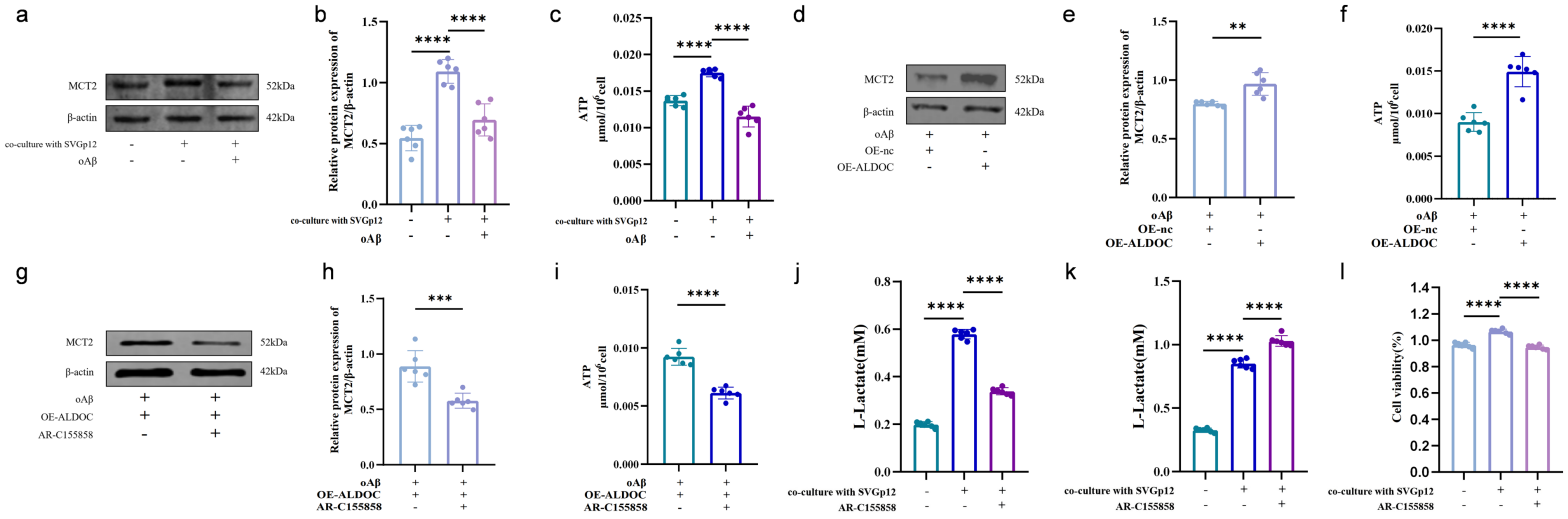
ALDOC overexpression in oAβ-treated astrocytes reprograms astrocytic metabolism and rescues neuronal energy deficits via lactate shuttling in an *in vitro* AD model. **(a)** Western blot image of MCT2 in SVGp12 cells co-cultured with SH-SY5Y cells. **(b)** Quantitative protein analysis of MCT2 in **(a)** (*n* = 6). **(c)** Changes in ATP content in SVGp12 cells cocultured with SH-SY5Y cells (*n* = 6). **(d)** Western blot image of MCT2 in SH-SY5Y cells co-cultured with SVGp12 cells after ALDOC overexpression. **(e)** Quantitative protein analysis of MCT2 in **(d)** (*n* = 6). **(f)** Changes in ATP content in SH-SY5Y cells cocultured with SVGp12 cells after ALDOC overexpression (*n* = 6). **(g)** Western blot image of MCT2 in SH-SY5Y cells co-cultured with ALDOC overexpressed SVGp12 cells with or without AR-C155858 intervention. **(h)** Quantitative analysis of MCT2 proteins in SH-SY5Y cells co-cultured with ALDOC overexpressed SVGp12 cells with or without AR-C155858 intervention (*n* = 6). **(i)** ATP content level of SH-SY5Y cells co-cultured with ALDOC overexpressed SVGp12 cells with or without AR-C155858 intervened (*n* = 6). **(j)** Changes in lactate levels in SH-SY5Y cells in co-culture system with SVGp12 cells (*n* = 6). **(k)** Changes in lactate levels in culture medium in the co-culture system with SVGp12 cells (*n* = 6). **(l)** Levels of cell viability in SH-SY5Y cells when co-cultured with SVGp12 cells (*n* = 6). **(a–l)** All experiments were performed independently using samples derived from at least three different batches (*n* ≥ 3 per group). *p* < 0.01, ***p < 0.001, ****p < 0.0001, ns (non-significant). Scale bars are indicated directly on the image.

*In vivo*, astrocytes play an indispensable role in providing energy support to neurons (Brandebura et al., 2023; Allen and Eroglu, 2017). To investigate this physiological relationship *in vitro*, we established a coculture system of SVGp12 astrocytes and SH-SY5Y neuronal cells (Supplementary Figure S4a). Compared with SH-SY5Y cells cultured alone, co-culture increased the expression of the lactate transporter MCT2 (*p* < 0.0001; Figures 5a,b) and the cellular ATP content (*p* < 0.0001; Figure 5c). However, pretreatment with oAβ reduced the expression of MCT2 (*p* < 0.0001; Figures 5a,b) and ATP (*p* < 0.0001; Figure 5c).

We next examined whether astrocytic ALDOC overexpression could counteract the oAβ-induced impairment in SVGp12 cells and thereby improve energy support to SH-SY5Y neurons. Indeed, ALDOC overexpression in oAβ-treated SVGp12 cells rescued MCT2 expression (*p* < 0.01; Figures 5d,e) and increased the total ATP content (*p* < 0.0001; Figure 5f) in the coculture system. These results suggest that ALDOC overexpression enhances glycolytic activity in oAβ-treated astrocytes, thereby increasing the energy supply to cocultured neurons. Immunofluorescence analysis corroborated these findings. Coculture with SVGp12 cells increased the fluorescence intensity of MCT2 (*p* < 0.05; Supplementary Figures S4b,c) in SH-SY5Y cells. While oAβ treatment of SVGp12 cells diminished this effect (MCT2, *p* < 0.01; Supplementary Figures S4b,c), ALDOC overexpression effectively restored the expression of MCT2 (*p* < 0.05; Supplementary Figures S4b,c).

To further investigate whether this energy support depends on the astrocyte-neuron lactate shuttle, we inhibited the activity of the neuronal lactate transporter MCT2 with AR-C155858. MCT2 is a primary lactate transporter on neurons and a core component of the astrocyte-neuron lactate shuttle. In the co-culture system, the application of AR-C155858 (3 μM, 2 h) effectively inhibited MCT2 expression in SH-SY5Y cells (*p* < 0.001; Figures 5g,h). Compared with no inhibitor treatment, MCT2 inhibition significantly decreased the ATP production (*p* < 0.0001; Figure 5i). AR-C155858 treatment induced a shift in lactate distribution in SH-SY5Y cells, characterized by decreased intracellular lactate levels (*p* < 0.0001; Figure 5j) and increased extracellular lactate levels (*p* < 0.0001; Figure 5k), along with reduced cell viability (*p* < 0.0001; Supplementary Figure Sl). Taken together, these results indicate that in the coculture model, ALDOC overexpression reverses oAβ-induced metabolic deficits in SVGp12 astrocytes by enhancing glycolysis. The resulting lactate appears to be shuttled to SH-SY5Y neurons via MCT2, contributing to energy support.

### Magnesium upregulates ALDOC expression, thereby promoting glycolytic flux in oAβ-treated astrocytes

**Figure 6:**
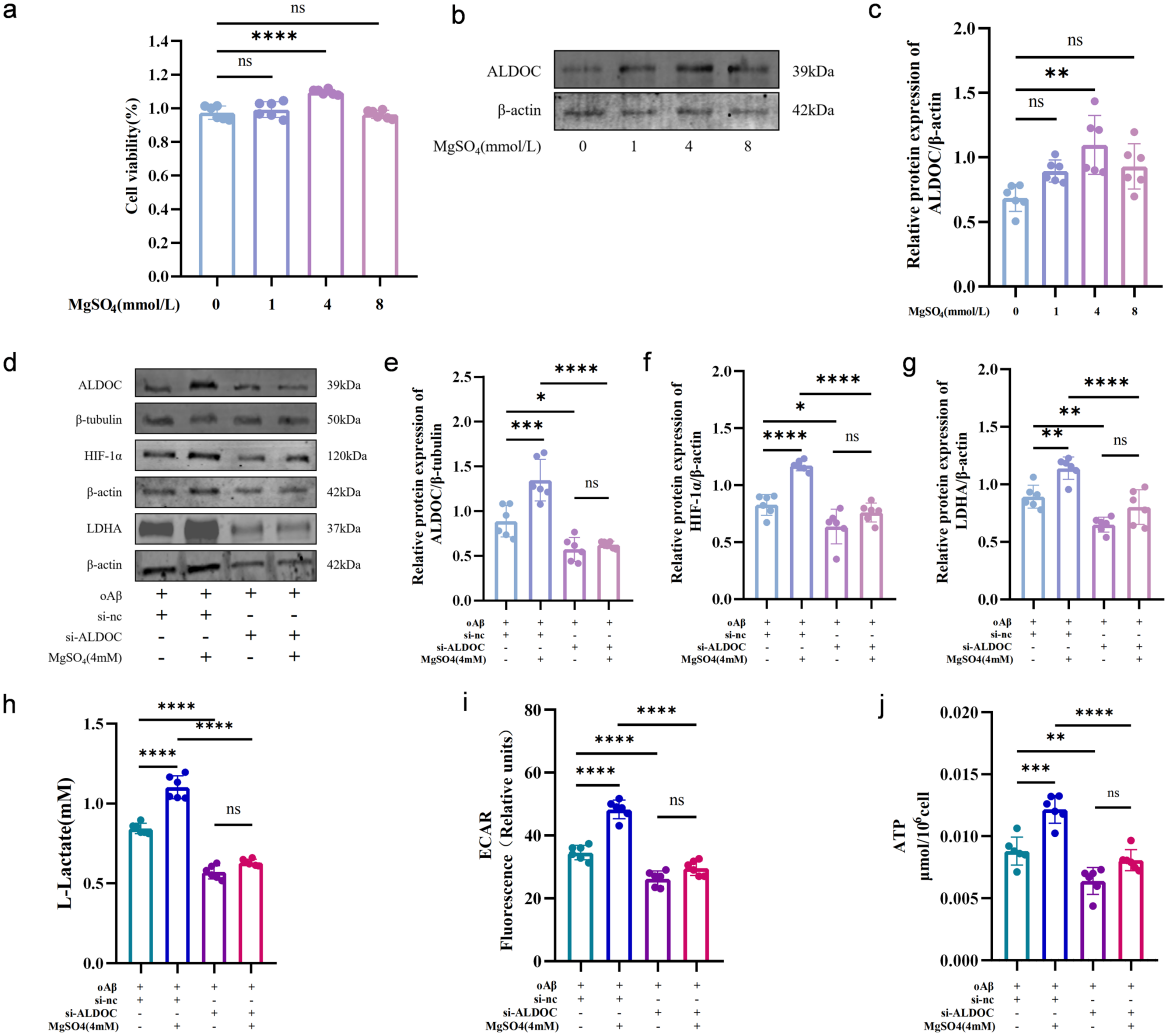
MgSO_4_ upregulated ALDOC expression in oAβ-treated-SVG p12 cells and enhanced glycolysis. **(a)** Viability of oAβ + SVG cells following 24 h treatment with varying concentrations of magnesium sulfate (*n* = 6). **(b)** Western blot image of ALDOC in oAβ + SVG cells after 24 h treatment with varying concentrations of magnesium sulfate. **(c)** Quantitative protein analysis **(b)** (*n* = 6). **(d)** Western blot image of ALDOC in oAβ + SVG cells with or without magnesium sulfate intervention, subjected to ALDOC knockdown. **(e–g)** Quantitative protein analysis **(d)** (*n* = 6). **(h–j)** Lactate levels, ECAR levels, and ATP content in oAβ + SVG cells with or without magnesium sulfate intervention, subjected to ALDOC knockdown (*n* = 6). **(a–j)** All experiments were performed independently three times using samples derived from at least three different batches (*n* = 6 per group); *p < 0.05, **p < 0.01, *p* < 0.001, ****p < 0.001, ns (non-significant).

Magnesium (Mg) is an essential cofactor for aldolases and regulates their expression (Salinas et al., 2023). To investigate whether Mg specifically or non-specifically enhanced ALDOC expression, SVGp12 cells were treated with magnesium sulfate (MgSO_4_). Based on physiological magnesium levels in normal cells and previous cellular studies (de Sánchez Muniain et al., 2025, Yu et al., 2010), cells were treated with 0, 1, 4, and 8 mM MgSO_4_ for 24 h. CCK-8 assays revealed that cell viability showed an increasing trend at 4 mM MgSO_4_ (*p* < 0.0001; Figure 6a). Western blot results showed that, compared with the 0 mM group, ALDOC protein expression did not change significantly upon treatment with 1 mM and 8 mM MgSO_4_ (*P* ˃ 0.05; Figures 6b,c). Treatment with 4 mM MgSO4 significantly increased ALDOC protein expression (*p* < 0.01; Figures 6b,c). Considering both protein expression and cell viability, 4 mM MgSO_4_ was selected for subsequent experiments, consistent with previous reports (de Sánchez Muniain et al., 2025; Yu et al., 2010).

Treatment with 4 mM MgSO_4_ significantly increased the expression of ALDOC (*p* < 0.001; Figures 6d, E) as well as that of its downstream effectors HIF-1α (*p* < 0.0001; Figures 6d,f) and LDHA (*p* < 0.01; Figures 6d,g). Concurrently, we observed elevated lactate (*p* < 0.0001, Figure 6h), ECAR (*p* < 0.0001, Figure 6i), and ATP (*p* < 0.001, Figure 6j) levels. To determine whether MgSO_4_ acts primarily through ALDOC, we knocked down ALDOC using siRNA prior to MgSO_4_ treatment. Subsequently, the expression levels of ALDOC (*p* < 0.05; Figures 6d,e), HIF-1α (*p* < 0.05; Figures 6d,f), and LDHA (*p* < 0.01; Figures 6d,g) decreased significantly. This decrease was consistent with the changes in glycolytic indicators, including lactate content (*p* < 0.0001, Figure 6h), ECAR (*p* < 0.0001, Figure 6i), and ATP content (*p* < 0.01, Figure 6j). Compared with the MgSO_4_ intervention group, the effects of MgSO_4_ were significantly reduced. After treatment of ALDOC-knockdown cells with 4 mM MgSO_4_, no significant upregulation was observed in the protein expression levels of ALDOC (*P* ˃ 0.05; Figures 6d,e), HIF-1α (*P* ˃ 0.05; Figures 6d,f), and LDHA (*P* ˃ 0.05; Figures 6d,g). Similarly, lactate levels (*P* ˃ 0.05, Figure 6h), ECAR (*P* ˃ 0.05, Figure 6i), and ATP content (*P* ˃ 0.05, Figure 6j) remained unchanged. These results suggest that 4 mM MgSO_4_ enhances ALDOC protein expression, which, in turn, promotes metabolic reprogramming and boosts glycolytic flux in SVGp12 cells. The attenuated effect of ALDOC knockdown indicates that this pro-glycolytic action may be mediated through ALDOC.

## Discussion

Our results revealed that ALDOC expression is reduced in reactive astrocytes in an AD mouse model and in oAβ-treated human astrocytes. This reduction is associated with decreased glycolytic activity and altered AMPK/mTOR/HIF-1α signaling. Gain- and loss-of-function experiments revealed that ALDOC modulates astrocytic glycolysis, at least in part, through HIF-1α. In a coculture system, ALDOC overexpression in astrocytes improved neuronal energy support, an effect that appeared to depend on the MCT2-mediated lactate shuttle. Furthermore, MgSO_4_ enhances ALDOC expression, leading to altered glycolytic levels in oAβ-treated astrocytes. Taken together, these findings suggest that ALDOC plays a role in astrocytic metabolic regulation and may influence neuronal function in the context of AD pathology. Further studies are needed to establish direct causality and to determine the therapeutic potential of targeting ALDOC.

Astrocytes, the most abundant glial cells in the brain, are pivotal for maintaining neuronal homeostasis by providing structural, metabolic, and trophic support (Rodríguez-Arellano et al., 2016; Sofroniew and Vinters, 2010). Under physiological conditions, they regulate synaptic plasticity, buffer neurotransmitters, and supply energy substrates such as lactate via glycolytic pathways, thereby supporting neuronal function and survival (Sofroniew and Vinters, 2010). The brain has a complex metabolic landscape that is finely adapted to the distinct functional roles of its cellular constituents (Traxler et al., 2021). Among brain metabolic pathways, glucose metabolism serves as a primary energy source for sustaining synaptic activity, ion homeostasis, and neurotransmitter synthesis (Shichkova et al., 2024). Neurons meet their high ATP demands mainly through mitochondrial oxidative phosphorylation, whereas astrocytes exhibit a distinct metabolic profile characterized by prominent glycolysis even under normoxic conditions (Chen Z. et al., 2023). This “glycolytic specialization” enables astrocytes to rapidly convert glucose into lactate, which is then exported to neurons via monocarboxylate transporters (MCTs) to fuel neuronal oxidative metabolism—a process termed the astrocyte–neuron lactate shuttle (ANLS) (Magistretti and Allaman, 2018). In neurodegenerative diseases such as AD, metabolic changes in astrocytes affect lactate shuttling, potentially contributing to neuronal bioenergetic deficits and neurotoxicity (Calì et al., 2024). Consistent with this notion, our study revealed the downregulation of key glycolytic factors, including ALDOC, HIF-1α, LDHA, and PKM2, in reactive astrocytes surrounding Aβ plaques and in astrocytes exposed to Aβ *in vitro*, suggesting altered glucose metabolism in these cells. Moreover, oAβ-treated astrocytes also exhibited significantly reduced GLT-1 expression, indicating impaired glutamate uptake capacity. Furthermore, *in vitro* astrocyte-neuron coculture experiments revealed that Aβ exposure reduced astrocytic lactate production, was associated with disruption of the ANLS, and was correlated with reduced neuronal function.

ALDOC belongs to the aldolase family, which regulates glycolysis and gluconeogenesis by reversibly catalyzing the cleavage of FBP into DHAP and G3P (Chang et al., 2018; Chen et al., 2014). Unlike ALDOA and ALDOB, which are involved primarily in glycolysis and fructose metabolism in muscle and liver, respectively, ALDOC is highly abundant in the central nervous system (CNS), particularly in forebrain astrocytes, Purkinje cells, and hippocampal cells (Chen et al., 2021; Tachikawa et al., 2004). Given that astrocytes are the principal energy-supporting cells for neurons, ALDOC may regulate astrocytic metabolic function. Recent studies in glioblastoma (GBM) have reported that hypermethylation-mediated silencing of ALDOC contributes to tumor progression by promoting invasion and migration and has been linked to serotonin hypersecretion and suppression of PPAR-*γ* signaling (Chang et al., 2024). Beyond its metabolic function, ALDOC possesses broad protein-binding properties and interacts directly with cytoskeletal components, suggesting its involvement in cytoskeletal rearrangement, intracellular transport, and signal transduction (Merkulova et al., 2011; Sandoval et al., 2013). Although evidence indicates that ALDOC expression is dysregulated in patients with neurodegenerative diseases, its precise mechanistic role remains unclear (Peng et al., 2024; Wang et al., 2021). In the present study, we observed reduced ALDOC expression in reactive astrocytes surrounding Aβ plaques, accompanied by altered AMPK/mTOR/HIF-1α signaling and downregulation of key glycolytic enzymes. Consistent with these *in vivo* findings, parallel metabolic changes were reproduced *in vitro* in oAβ-treated astrocytes. Notably, the overexpression of ALDOC in oAβ-exposed astrocytes was associated with a restored p-AMPK/p-mTOR/HIF-1α balance and increased expression of the glycolytic enzymes LDHA and PKM2. Together, these findings suggest that ALDOC regulates astrocytic metabolism in AD, possibly by modulating AMPK/mTOR/HIF-1α signaling.

AMPK is a key sensor of cellular energy and nutrient status and is activated under low-energy conditions, in turn promoting homeostatic responses (Lin and Hardie, 2018; Daval et al., 2006; Davies et al., 1995). Its activation occurs primarily through two distinct mechanisms: one involves canonical AMP/ADP-dependent signaling under energy stress, whereas the other involves a noncanonical, nutrient-sensing pathway responsive to factors such as glucose availability (Lin and Hardie, 2018; Daval et al., 2006; Davies et al., 1995). In the latter pathway, activation can be triggered when an individual senses a decrease in its substrate FBP; under glucose deprivation, reduced FBP levels promote AMPK activation (Zhang et al., 2017). Since its discovery, glucose deprivation has been widely used in cell culture to robustly activate AMPK (Salt et al., 1998). Previous *in vitro* studies have shown that oAβ exposure impairs cellular glucose utilization and disrupts mitochondrial function, thereby inducing a state of functional hypoglycemia in astrocytes (Brandebura et al., 2023; Zheng et al., 2021). This approach may more closely mimic the pathological metabolic environment observed in AD patients and animal models than simple glucose withdrawal does. In our experimental model, we treated astrocyte cell lines with oAβ. Moreover, JC-1 staining revealed that oAβ treatment significantly reduced the mitochondrial membrane potential in astrocytes, indicating mitochondrial dysfunction beyond glycolytic impairment.

Emerging evidence indicates that AMPK plays a complex and cell-type-specific role in AD pathogenesis (Daval et al., 2006; Shukitt-Hale et al., 2015). While acute AMPK activation can be adaptive, chronic or excessive AMPK signaling may paradoxically increase Aβ production by upregulating BACE1 activity and exacerbating tau pathology through direct phosphorylation (Chen et al., 2009; Salminen et al., 2011). In astrocytes, sustained AMPK activation can alter glycolytic flux and lactate production, potentially compromising the astrocyte-neuron lactate shuttle and, in turn, the neuronal energy supply (Chen W. et al., 2023; Wang et al., 2025). AMPK activation inhibits mTOR activity through both direct and indirect mechanisms. AMPK indirectly phosphorylates and activates tuberous sclerosis complex 2 (TSC2), a negative regulator of the mTORC1 complex. AMPK can also directly phosphorylate Raptor, a key component of mTORC1, thereby suppressing mTORC1. mTOR itself is critically involved in AD pathogenesis and regulates processes such as autophagy, neuroinflammation, Aβ and tau pathology, and metabolic homeostasis. Notably, under AD-related glucose deprivation, microglia shift toward glycolysis, which can exacerbate neuroinflammation; mTOR inhibition has been shown to restore oxidative phosphorylation and attenuate this inflammatory response (Yang et al., 2023). However, the role of mTOR in astrocytes is distinct because it relies on glycolysis for ATP production even under aerobic conditions. Specifically, the mTORC1 complex promotes HIF-1α protein synthesis by enhancing its translation, whereas mTOR inhibition reduces HIF-1α expression and thereby suppresses downstream glycolytic enzymes (Shang et al., 2024; Wang H. et al., 2024; Wang Y. et al., 2024). HIF-1α serves as a master transcriptional regulator of cellular adaptation to hypoxia and, importantly, upregulates a suite of glycolytic enzymes to sustain energy production under low-oxygen or metabolic stress conditions (Zhang et al., 2021; Chen et al., 2018). In AD, cerebral glucose metabolism is severely impaired. HIF-1α, a core transcription factor that is responsive to cellular energy stress, upregulates the expression of genes encoding many glucose transporters and glycolytic enzymes. This response promotes a metabolic shift from oxidative phosphorylation to glycolysis, enabling rapid ATP production during an energy crisis and helping to sustain basic CNS function. As the disease progresses, however, the accumulation of Aβ and tau pathology inhibits glycolytic flux, and HIF-1α expression consequently tends to decrease (van Gijsel-Bonnello et al., 2017; Ashok et al., 2017). In advanced disease stages, appropriately elevating HIF-1α levels in astrocytes to increase glycolysis represents a potential therapeutic strategy worthy of further exploration. In this study, we observed significant downregulation of HIF-1α and key glycolytic enzymes in reactive astrocytes from an AD mouse model. Consistent with these findings, *in vitro* experiments revealed that oAβ-treated astrocytes exhibited decreased ALDOC expression, altered AMPK/mTOR signaling, and reduced HIF-1α and glycolytic enzyme levels. Modulation of ALDOC expression was associated with corresponding changes in the AMPK/mTOR/HIF-1α axis. Importantly, rescue experiments revealed that knocking down HIF-1α expression in ALDOC-overexpressing astrocytes prevented the recovery of PKM2 and LDHA expression and failed to restore glycolytic function. Notably, the increased p-AMPK/AMPK ratio observed in oAβ-treated astrocytes likely reflects a compensatory energy stress response, whereas the reduction in this ratio upon ALDOC overexpression suggests the restoration of cellular energy homeostasis rather than a direct inhibitory effect on AMPK. Together, these results suggest that ALDOC regulates astrocytic glucose metabolism in AD, possibly through the AMPK/mTOR/HIF-1α pathway.

MCT2, a neuron-specific member of the monocarboxylate transporter family, is predominantly localized to postsynaptic membranes and axons. It mediates the uptake of astrocyte-derived lactate into neurons, a key step in the astrocyte-neuron lactate shuttle that supplies energy for neuronal activity (Guan et al., 2019). In AD, significantly reduced MCT2 expression impairs neuronal lactate uptake. This energy deficit may compromise synaptic plasticity and neurotransmitter recycling, potentially contributing to cognitive decline (Zhang et al., 2018). Whether restoring MCT2 function could slow AD progression requires further investigation. In the present study, we found that ALDOC overexpression in astrocytes increased extracellular lactate levels and was associated with upregulated neuronal MCT2 expression and increased neuronal activity. Conversely, inhibition of MCT2 abrogated this protective effect. Together, these results suggest that, in the context of AD, ALDOC-associated metabolic changes in astrocytes may increase lactate production, which in turn could support neurons via the lactate shuttle, thereby contributing to improved neuronal function.

Magnesium (Mg) is an essential cofactor for the catalytic mechanisms of aldolases. In Class II aldolases, including ALDOC, Mg is indispensable for polarizing the carbonyl group of the substrate to facilitate nucleophilic attack during carbon–carbon bond formation. Simultaneously, it stabilizes key reaction intermediates and helps maintain the structural integrity of the enzyme’s active site (Salinas et al., 2023). Recent studies on engineered aldolases, such as PLP-dependent UstD, have demonstrated that metal-ion coordination can influence regioselectivity and stereoselectivity, enabling the synthesis of diverse chiral imino acids with high efficiency (up to 94% selectivity) (Lan et al., 2025). Additionally, Mg deficiency has been linked to metabolic dysregulation, as aldolases are key enzymes in glycolysis and gluconeogenesis, with aberrant activity associated with diseases, such as hepatocellular carcinoma (Salinas et al., 2023). In this study, we treated SVGp12 astrocytes with varying concentrations of MgSO_4_. These results demonstrated that 4 mM MgSO_4_ concentrations enhanced ALDOC expression. However, we noted that this 4 mM MgSO_4_ concentration used in the *in vitro* experiments exceeded normal serum magnesium levels. In a parallel experiment, we treated APP/PS1 mice with magnesium threonate (MgT) to further investigate the effective concentration of magnesium ions *in vivo*. When oA*β*-stimulated astrocytes were treated, ALDOC expression was higher than in the untreated group. Concurrently, upregulation of HIF-1α and the key glycolytic enzyme LDHA was detected, along with improved cell viability. However, ALDOC knockdown abolished some of the protective effects of MgSO_4_. Our preliminary study indicates that, *in vitro* cell experiments, MgSO_4_ can enhance ALDOC expression and promote the glycolytic capacity of astrocytes in the context of AD. However, whether this effect applies to *in vivo* models requires further investigation.

Several limitations of this study should be acknowledged. First, while our findings demonstrate an association between ALDOC expression and altered AMPK/mTOR/HIF-1α signaling, they do not establish direct causality. The observed changes in the p-AMPK/AMPK ratio following ALDOC manipulation likely reflect normalization of chronic energy stress rather than direct activation of the pathway. Future studies using genetic or pharmacological approaches with temporal control are needed to determine whether ALDOC directly regulates AMPK activity or acts indirectly through metabolic intermediates. Second, our experiments were conducted using an immortalized human astrocyte cell line (SVGp12) and a neuroblastoma cell line (SH-SY5Y). Although these cell lines provide a consistent and reproducible system for high-throughput studies, they exhibit altered basal metabolic rates compared to primary cells. Notably, both the immortalized astrocyte line and the cancer-derived SH-SY5Y cells inherently rely on aerobic glycolysis (the Warburg effect), which may not fully recapitulate the physiological and metabolic properties of primary astrocytes and neurons in the context of the astrocyte-neuron lactate shuttle. Therefore, validation of our key findings in primary murine astrocyte-neuron co-cultures, or in human iPSC-derived co-culture systems, and *in vivo* with astrocyte-specific ALDOC manipulation is needed. Third, as an exploratory study, the sample size in some experiments was relatively limited. Nonetheless, the observed differences were statistically significant and consistent across multiple assays. Larger-scale studies would help to further strengthen the conclusions. Finally, the potential role of magnesium was not mechanistically investigated. Given its incomplete characterization, it warrants further investigation.

In summary, AD is a neurodegenerative disorder associated with cerebral metabolic dysregulation, in which metabolic changes in astrocytes may play a role. Our results revealed that ALDOC expression is downregulated in APP/PS1 mice, accompanied by altered AMPK/mTOR/HIF-1α signaling and impaired glycolysis. These changes may reduce the capacity of astrocytes to provide metabolic support to neurons. *In vitro* studies further revealed that modulating ALDOC expression influences AMPK/mTOR/HIF-1α signaling and glycolytic flux, which in turn may affect neuronal function through the lactate shuttle. Notably, MgSO_4_ rescued ALDOC-mediated metabolic alterations in astrocytes, highlighting its potential as a therapeutic strategy for AD. Together, these findings suggest a potential metabolic mechanism involving ALDOC in AD pathogenesis and may offer possible targets for further investigation.

## Data Availability

The raw data supporting the conclusions of this article will be made available by the authors, without undue reservation.

## Supplementary material

SUPPLEMENTARY FIGURE S1 Immunofluorescence images of the hippocampus of APP/PS1 mice and WT mice. **(a–d)** Representative immunofluorescence image of ALDOC, HIF-1α, and LDHA in the hippocampus of WT mice and APP/PS1 transgenic mice, and quantitative fluorescence analysis (*n* = 3). **(e–h)** Representative immunofluorescence image of ALDOC, HIF-1α, and LDHA in the cortex of WT mice and APP/PS1 transgenic mice, and quantitative fluorescence analysis (*n* = 3). **(i)** Representative immunofluorescence image of NEUN and ALDOC (double-staining) in the hippocampus of APP/PS1 transgenic mice. **(j)** Fluorescence colocalization scatter plot of ALDOC and GFAP in the hippocampus of APP/PS1 mice. **(k)** Fluorescence colocalization scatter plot of ALDOC and NEUN in the hippocampus of APP/PS1 mice. ***p* < 0.01, ****p* < 0.001, ns (non-significant). Scale bars are indicated directly on the image.

SUPPLEMENTARY FIGURE S2 oAβ intervention significantly decreased the expression levels of ALDOC, HIF-1α, and LDHA in SVGp12 cells. (a-d)Representative immunofluorescence image of ALDOC, HIF-1α, and LDHA in none-intervened SVGp12 cells and oAβ-intervened SVGp12 cells, and quantitative fluorescence analysis (*n*=3). **p* < 0.05, ns (non-significant). Scale bars are indicated directly on the image.

SUPPLEMENTARY FIGURE S3 Down-regulating ALDOC expression in SVGp12 cells followed by oAβ intervention consequently altered the expression of the AMPK-mTOR-HIF1α pathway and affected glycolytic levels. **(a)** Knockdown efficiency of ALDOC gene by different siRNA (*n* = 6). **(b)** Western blot image of ALDOC, pAMPK, AMPK, pMTOR, mTOR, HIF-1α, LDHA, and PKM2 in oAβ + SVGp12 cells with ALDOC gene knockdown or none knockdown. **(c–h)** Quantitative protein analysis of ALDOC, pAMPK, AMPK, pMTOR, mTOR, HIF-1α, LDHA, PKM2 in (P) (*n* = 6). **(i–k)** Relative mRNA expression levels of HIF-1α, LDHA, PKM2 in oAβ + SVGp12 cells following ALDOC knockdown (*n* = 6). **(l–n)** L-lactate level, ECAR level, ATP content in oAβ + SVGp12 cells following ALDOC knockdown (*n* = 6). **(a–n)** All experiments were performed independently using samples derived from at least six different batches (*n* = 6 per group) **p* < 0.05, ***p* < 0.01, ****p* < 0.001, *****p*<0.0001, ns (non-significant).

SUPPLEMENTARY FIGURE S4 ALDOC-driven glycolytic activation in oAβ-treated astrocytes enhanced synaptic function of neurons. **(a)** SVGp12 cells (lower chamber) were co-cultured with SH-SY5Y (upper chamber) via transwell apparatus. **(b)** Representative immunofluorescence image of MCT2 in SH-SY5Y cells under different conditions of oAβ intervention and overexpression of ALDOC in SVGp12 cells and SH-SY5Y cell co-culture systems. And statistical analysis of MCT2 fluorescence intensity in **(c)** (*n* = 3). **(b,c)** All experiments were performed independently using samples derived from three different batches (*n* = 3 per group). **p* < 0.05, ***p* < 0.01, ns (non-significant).

## Glossary

AD: Alzheimer’s disease
ALDOC: aldolase C
WT: wild type
oAβ: oligomeric amyloid-β
ECAR: extracellular acidification rate
HIF-1α: hypoxia-inducible factor-1α
Aβ: *β*-amyloid
NFTs: neurofibrillary tangles
TCA cycle: tricarboxylic acid cycle
OXPHOS: oxidative phosphorylation
ATP: adenosine triphosphate
AG: aerobic glycolysis
G3P: glyceraldehyde-3-phosphate
DHAP: dihydroxyacetone phosphate
CNS: central nervous system
PD: Parkinson’s disease
AMP: adenosine monophosphate
AMPK: AMP-activated protein kinase
mTOR: mechanistic target of rapamycin
FBP: fructose-1,6-bisphosphate
LDHA: lactate dehydrogenase A
PKM2: pyruvate kinase M2
IACUC: Institutional Animal Care and Use Committee
HFIP: 1,1,1,3,3,3-hexafluoro-2-propanol
CCK-8: cell counting kit
IFD: integrated fluorescence density
SEM: standard error of mean
ANOVA: analysis of variance
GLT-1: glutamate transporter-1
MCTs: monocarboxylate transporters
ANLS: astrocyte-neuron lactate shuttle
GBM: glioblastoma
TSC2: tuberous sclerosis complex 2
p-AMPK: phosphorylated-AMPK
P-mTOR: phosphorylated-mTOR
