# Supplementary figures and images for: ALDOC modulates astrocytic glycolysis and AMPK/mTOR/HIF-1α signaling in Alzheimer’s disease
Source: Front Neurosci. 2026 Jun 4;20:1847340. doi: 10.3389/fnins.2026.1847340 (PMC13275364; doi:10.3389/fnins.2026.1847340)

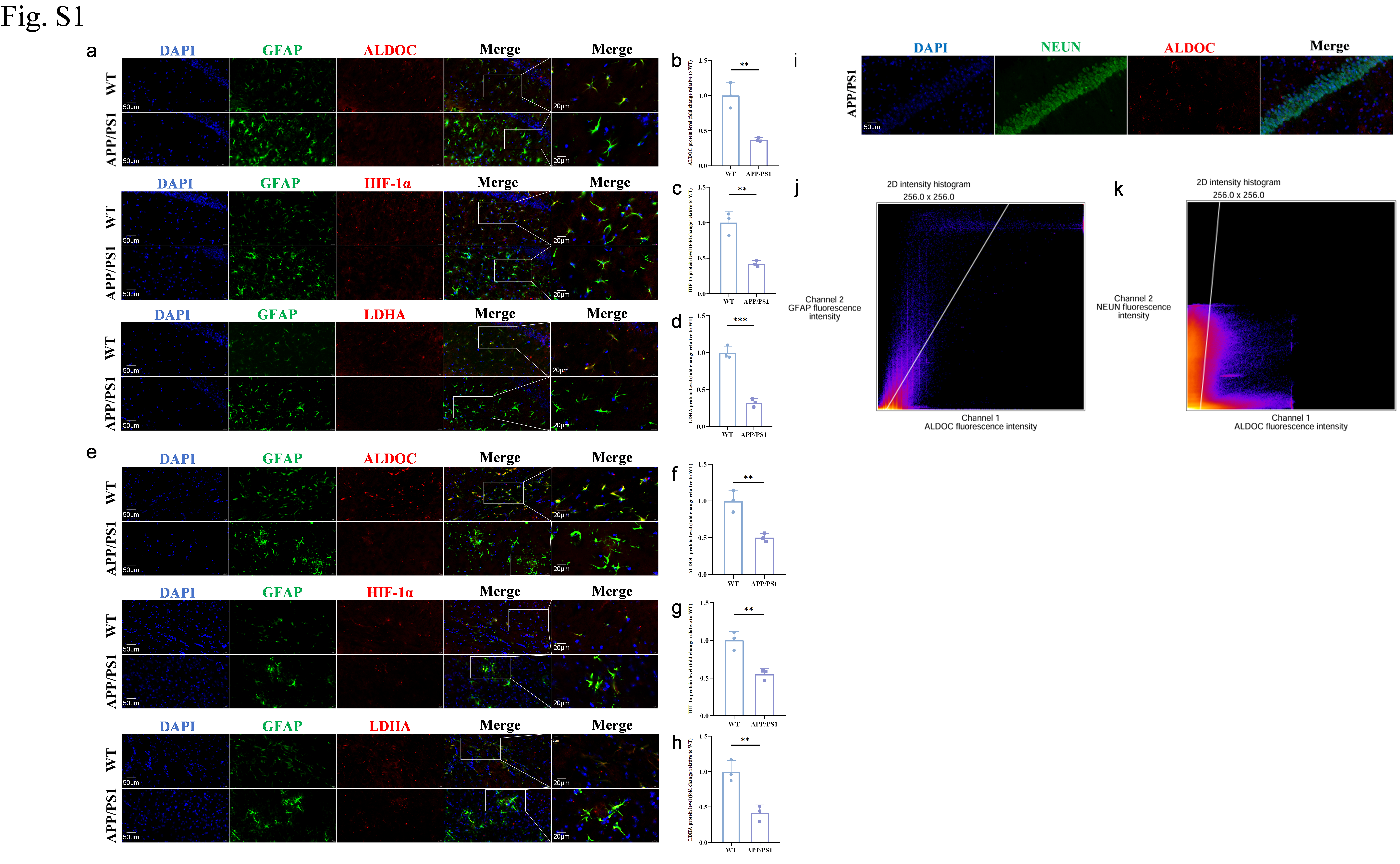

Supplement: SUPPLEMENTARY FIGURE S1 — Immunofluorescence images of the hippocampus of APP/PS1 mice and WT mice. (a–d) Representative immunofluorescence image of ALDOC, HIF-1α, and LDHA in the hippocampus of WT mice and APP/PS1 transgenic mice, and quantitative fluorescence analysis (n = 3). (e–h) Representative immunofluorescence image of ALDOC, HIF-1α, and LDHA in the cortex of WT mice and APP/PS1 transgenic mice, and quantitative fluorescence analysis (n = 3). (i) Representative immunofluorescence image of NEUN and ALDOC (double-staining) in the hippocampus of APP/PS1 transgenic mice. (j) Fluorescence colocalization scatter plot of ALDOC and GFAP in the hippocampus of APP/PS1 mice. (k) Fluorescence colocalization scatter plot of ALDOC and NEUN in the hippocampus of APP/PS1 mice. **p < 0.01, ***p < 0.001, ns (non-significant). Scale bars are indicated directly on the image. [file Image_1.TIF]

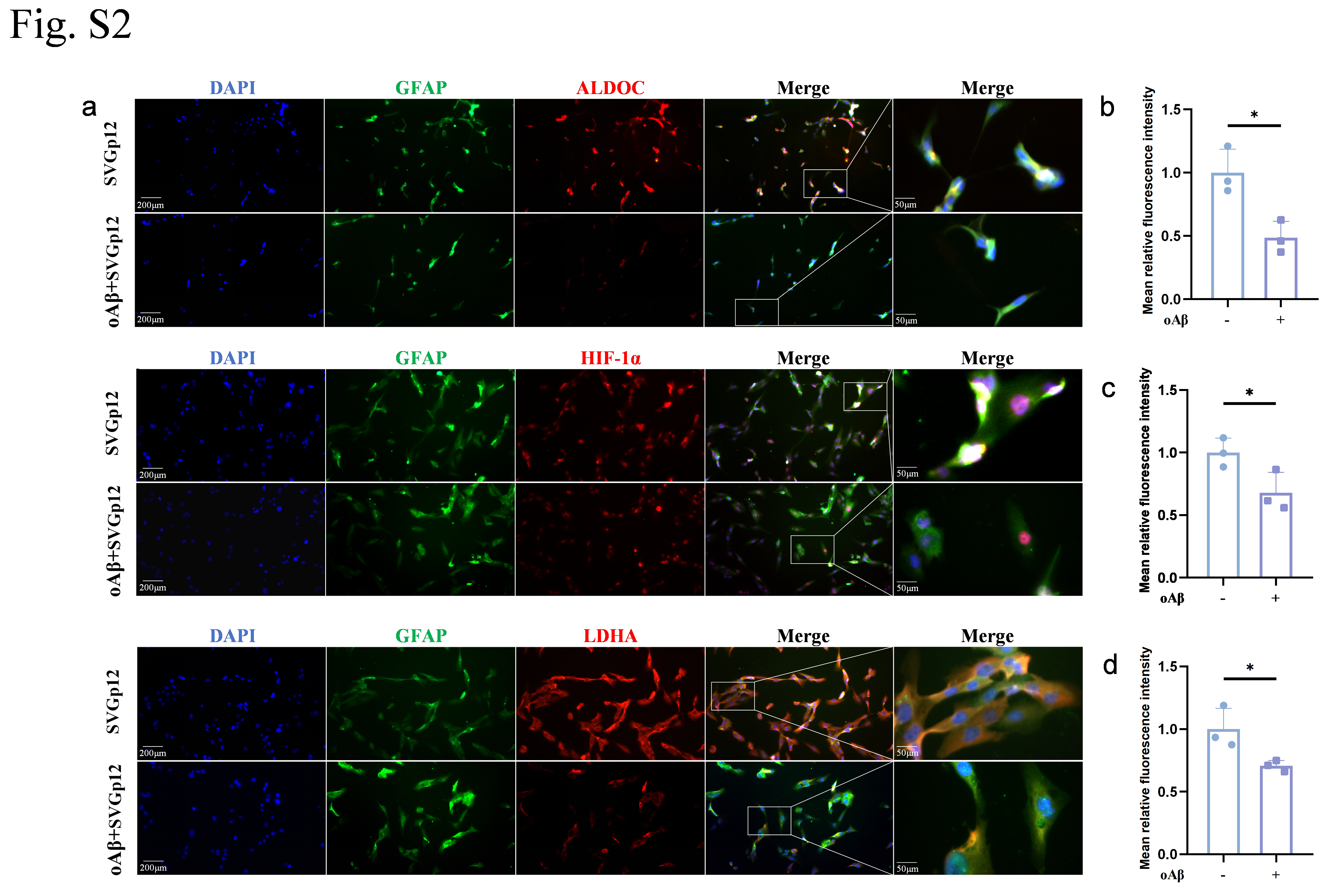

Supplement: SUPPLEMENTARY FIGURE S2 — oAβ intervention significantly decreased the expression levels of ALDOC, HIF-1α, and LDHA in SVGp12 cells. (a-d)Representative immunofluorescence image of ALDOC, HIF-1α, and LDHA in none-intervened SVGp12 cells and oAβ-intervened SVGp12 cells, and quantitative fluorescence analysis (n=3). *p < 0.05, ns (non-significant). Scale bars are indicated directly on the image. [file Image_2.TIF]

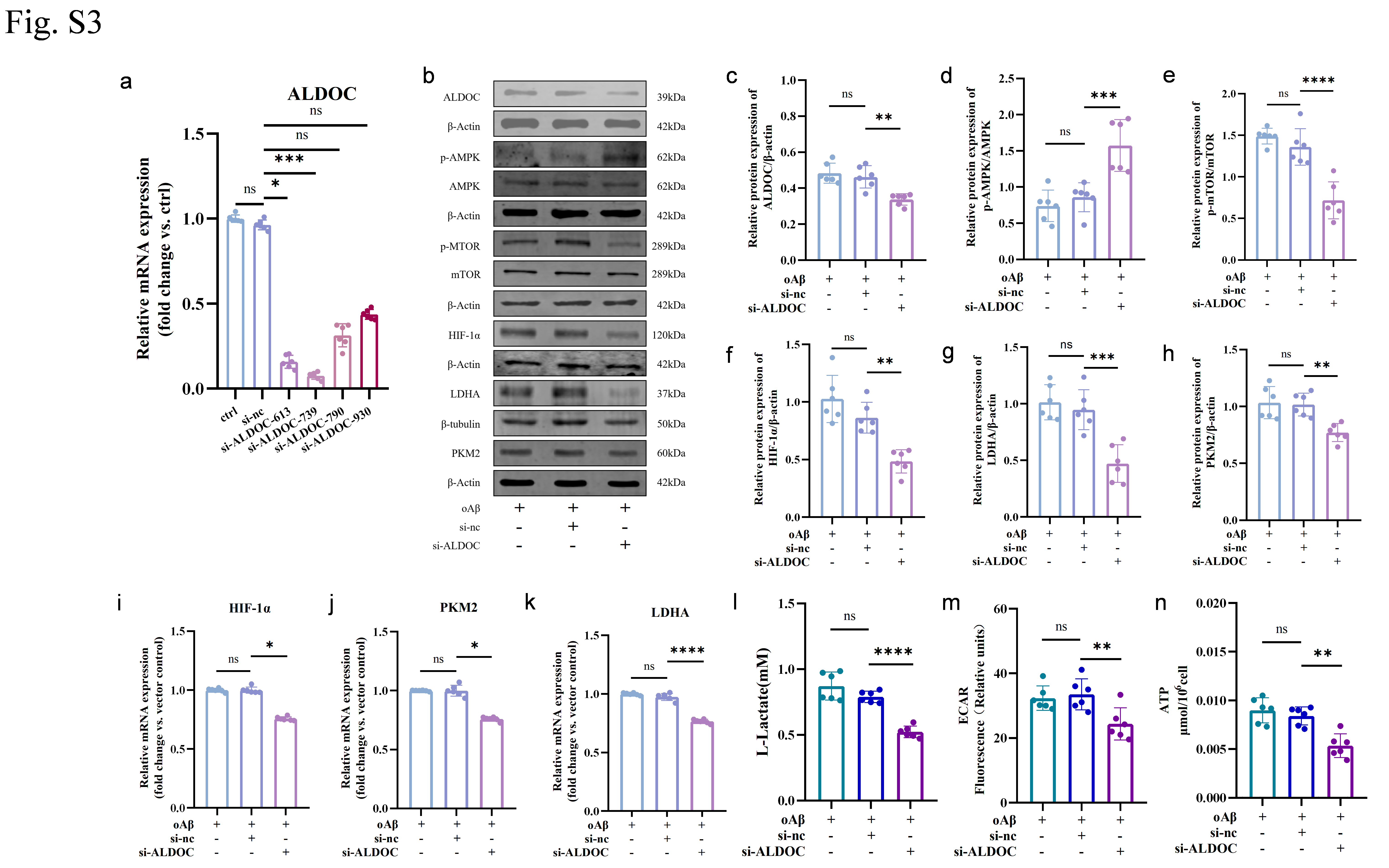

Supplement: SUPPLEMENTARY FIGURE S3 — Down-regulating ALDOC expression in SVGp12 cells followed by oAβ intervention consequently altered the expression of the AMPK-mTOR-HIF1α pathway and affected glycolytic levels. (a) Knockdown efficiency of ALDOC gene by different siRNA (n = 6). (b) Western blot image of ALDOC, pAMPK, AMPK, pMTOR, mTOR, HIF-1α, LDHA, and PKM2 in oAβ + SVGp12 cells with ALDOC gene knockdown or none knockdown. (c–h) Quantitative protein analysis of ALDOC, pAMPK, AMPK, pMTOR, mTOR, HIF-1α, LDHA, PKM2 in (P) (n = 6). (i–k) Relative mRNA expression levels of HIF-1α, LDHA, PKM2 in oAβ + SVGp12 cells following ALDOC knockdown (n = 6). (l–n) L-lactate level, ECAR level, ATP content in oAβ + SVGp12 cells following ALDOC knockdown (n = 6). (a–n) All experiments were performed independently using samples derived from at least six different batches (n = 6 per group) *p < 0.05, **p < 0.01, ***p < 0.001, ****p<0.0001, ns (non-significant). [file Image_3.TIF]

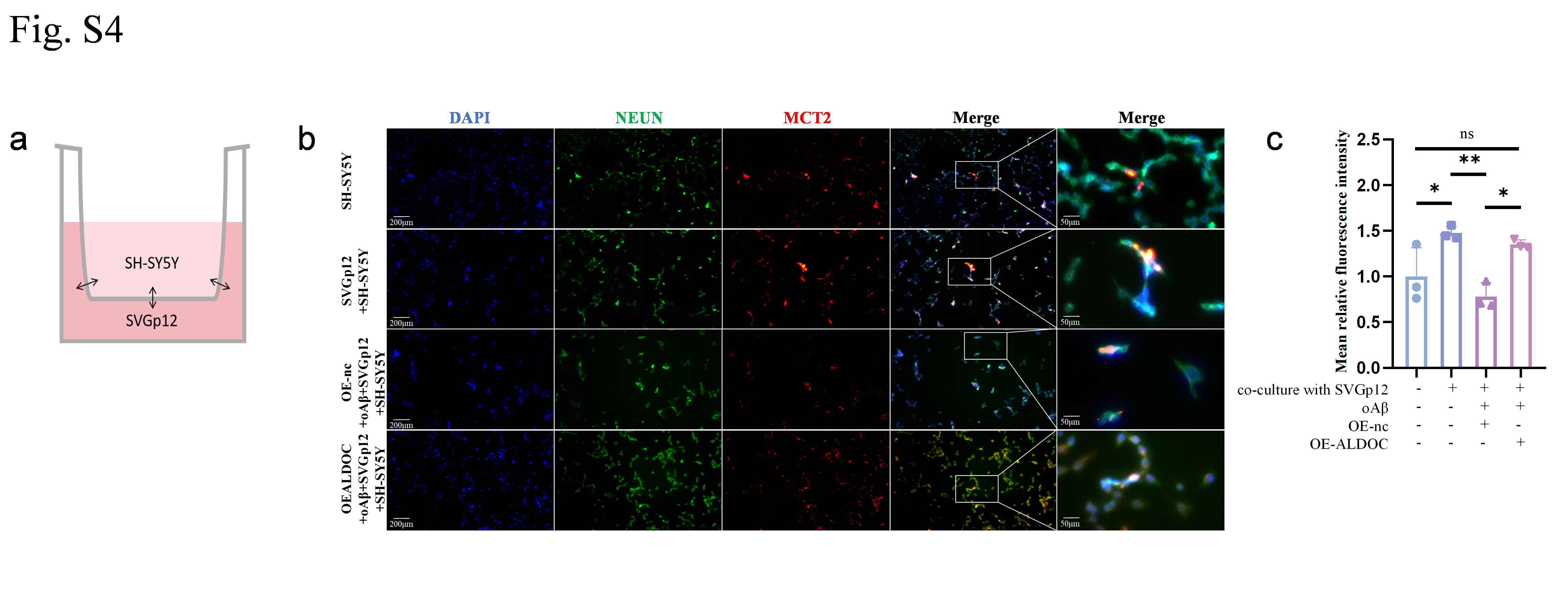

Supplement: SUPPLEMENTARY FIGURE S4 — ALDOC-driven glycolytic activation in oAβ-treated astrocytes enhanced synaptic function of neurons. (a) SVGp12 cells (lower chamber) were co-cultured with SH-SY5Y (upper chamber) via transwell apparatus. (b) Representative immunofluorescence image of MCT2 in SH-SY5Y cells under different conditions of oAβ intervention and overexpression of ALDOC in SVGp12 cells and SH-SY5Y cell co-culture systems. And statistical analysis of MCT2 fluorescence intensity in (c) (n = 3). (b,c) All experiments were performed independently using samples derived from three different batches (n = 3 per group). *p < 0.05, **p < 0.01, ns (non-significant). [file Image_4.TIF]
